# Long Term Effectiveness on Prescribing of Two Multifaceted Educational Interventions: Results of Two Large Scale Randomized Cluster Trials

**DOI:** 10.1371/journal.pone.0109915

**Published:** 2014-10-17

**Authors:** Nicola Magrini, Giulio Formoso, Oreste Capelli, Emilio Maestri, Francesco Nonino, Barbara Paltrinieri, Cinzia Del Giovane, Claudio Voci, Lucia Magnano, Lisa Daya, Anna Maria Marata, on behalf of the INDRA-NET study group

**Affiliations:** 1 Drug Evaluation Area, Emilia-Romagna Regional Agency for Health and Social Care, Bologna, Italy; 2 Local Health Authority, Modena, Italy; 3 Department of Clinical and Diagnostic Medicine and Public Health, University of Modena and Reggio Emilia, Modena, Italy; University of British Columbia, Canada

## Abstract

**Introduction:**

Information on benefits and risks of drugs is a key element affecting doctors’ prescribing decisions. Outreach visits promoting independent information have proved moderately effective in changing prescribing behaviours.

**Objectives:**

Testing the short and long-term effectiveness on general practitioners’ prescribing of small groups meetings led by pharmacists.

**Methods:**

Two cluster open randomised controlled trials (RCTs) were carried out in a large scale NHS setting. Ad hoc prepared evidence based material were used considering a therapeutic area approach - TEA, with information materials on osteoporosis or prostatic hyperplasia - and a single drug oriented approach - SIDRO, with information materials on me-too drugs of 2 different classes: barnidipine or prulifloxacin. In each study, all 115 Primary Care Groups in a Northern Italy area (2.2 million inhabitants, 1737 general practitioners) were randomised to educational small groups meetings, in which available evidence was provided together with drug utilization data and clinical scenarios. Main outcomes were changes in the six-months prescription of targeted drugs. Longer term results (24 and 48 months) were also evaluated.

**Results:**

In the TEA trial, one of the four primary outcomes showed a reduction (prescription of alfuzosin compared to tamsulosin and terazosin in benign prostatic hyperplasia: prescribing ratio −8.5%, p = 0.03). Another primary outcome (prescription of risedronate) showed a reduction at 24 and 48 months (−7.6%, p = 0.02; and −9,8%, p = 0.03), but not at six months (−5.1%, p = 0.36). In the SIDRO trial both primary outcomes showed a statistically significant reduction (prescription of barnidipine −9.8%, p = 0.02; prescription of prulifloxacin −11.1%, p = 0.04), which persisted or increased over time.

**Interpretation:**

These two cluster RCTs showed the large scale feasibility of a complex educational program in a NHS setting, and its potentially relevant long-term impact on prescribing habits, in particular when focusing on a single drug. National Health systems should invest in independent drug information programs.

**Trial Registration:**

Controlled-Trials.com ISRCTN05866587

## Introduction

Information on benefits and risks of drug treatments is an important element affecting doctors’ prescribing decisions though other factors such as clinical experience and therapeutic traditions, opinion leaders and local context influence, and also information coming from drug industry, play a role. Drug companies invest large sums in informing doctors through a wide range of activities: [1] promotion through drug representatives, sponsored events and symposia, and selective presentation of research findings,[2]–[5] which seem useful to the purpose of increasing sales, as companies spend on average a quarter of their revenues on marketing activities, almost twice as much as what they invest on research and development.[6]–[7].

As an international independent expert in drug policy expressed a few years ago “*a major challenge that remains is to reduce the imbalance between promotional and research expenditure, perhaps moving progressively towards a system in which health systems themselves provide the bulk of new drug information rather than indirectly funding, as they presently do, an army of company salesmen*”. [8] Access to the best available information (critically appraised, made easy and brought into small groups) is an important step towards a more balanced view of the actual benefits and risks of drugs allowing a more realistic estimation of the magnitude of the clinically relevant outcomes. Educational outreach visits have been proposed since 1983 as an effective method to promote change in prescribing practices, [9] using either one-to-one meetings with prescribers [10] or group meetings. [11] These methods have been subsequently implemented in various countries, [12] either to provide information from research papers and systematic reviews, free of recommendations, or to favour the use of clinical practice guidelines. Though information is mostly presented in relative terms (RR, OR, HR) there is an increasing tendency of the evidence-based movement towards favouring a presentation of trials results using actual benefits and absolute differences, possibly taking patients baseline risk into account.[13]–[14] The GRADE approach represents a good example in this regard. [15].

Available evidence on the effectiveness of outreach visits shows variable results, indicating modest to moderate effects (with relatively short-term assessments at 6–12 months), mostly assessing the implementation of clinical practice guidelines. [16]–[17] There is paucity of evidence formally evaluating the feasibility on a large scale basis of implementing independent information programs, and their long-term prescribing impact – up to several years. Moreover, although specific methods to put these principles into practice have been well conceptualized since about thirty years ago, [10] they have been often applied to academic detailing settings or to single-doctor meetings.

We applied these general principles and methods to a NHS drug information program consisting of twice-a-year small group sessions led by a pharmacist, established in several provinces of the Emilia-Romagna region since 2001. We avoided clinical practice guidelines as supporting material to drive behaviour-change since they were frequently perceived as cost-containment tools. [18].

Two pragmatic cluster randomised trials designed within the same protocol were designed and funded within a public research program.[19]–[20] Specific aims were to evaluate, in the short and long term, whether an intervention carried out in a natural' NHS setting, consisting of small group meetings of all general practitioners (the clusters), using clear and appealing educational materials, may change doctors’ prescribing behaviour. The two trials tested two different approaches: either relatively articulated and rich materials targeting a whole therapeutic area, or more focused materials targeting a newly approved single drug with rapidly increasing prescribing patterns.

## Methods

Two consecutive cluster trials, stemming from the same study protocol, were carried out in spring 2007 and autumn-winter 2007–08, respectively, in five Provinces of the Emilia Romagna region (Bologna, Forlì, Modena, Parma and Reggio Emilia, totalling about 2,200,000 inhabitants) involving all practising NHS General Practitioners. [21].

It was initially planned to have also the participation of 40 primary care groups from another region in Northern Italy, Friuli Venezia Giulia (about 400 GPs corresponding to an assisted population of about 440,000 inhabitants), but no agreement was reached between the Regional Health Authority and general practitioners’ organisations. For similar reasons, a parallel trial which had been planned in Sardinia was not implemented; such trial aimed to evaluate the effectiveness of different information formats delivered to single general practitioners and discussed by a pharmacist.

In order to evaluate and quantify the impact of such a program, we designed these randomised trials according to the following features:

- improving access to evidence-based clinically relevant information in a NHS setting using available resources;
- helping to assess the relevance and the limits of the available evidence, highlighting the role of systematic reviews and pitfalls due to publication bias, and also critically appraising the most relevant single studies;
- presenting benefit and risks clearly and effectively, [14] to provide better insight on their impact on clinical practice compared to market-driven interpretations from the drug industry; [22]
- integrating information from published studies with assessments from drug regulatory agencies [23] and other relevant contextual information;
- keeping in mind all the organisational factors and settings of what works in changing doctors’ behaviour: audit and feedback using prescribing data; [24] strengthening the role of pharmacists as facilitators; [16] favouring active learning in small groups. [12]

We identified doctors’ knowledge and attitudes as the main determinants of their prescription; therefore, we used the available resources to address these determinants through a dedicated information program. Specifically, the main principle embraced for our information campaign was the motto “doctor, we give you all the information, you decide”.

**Figure 1:**
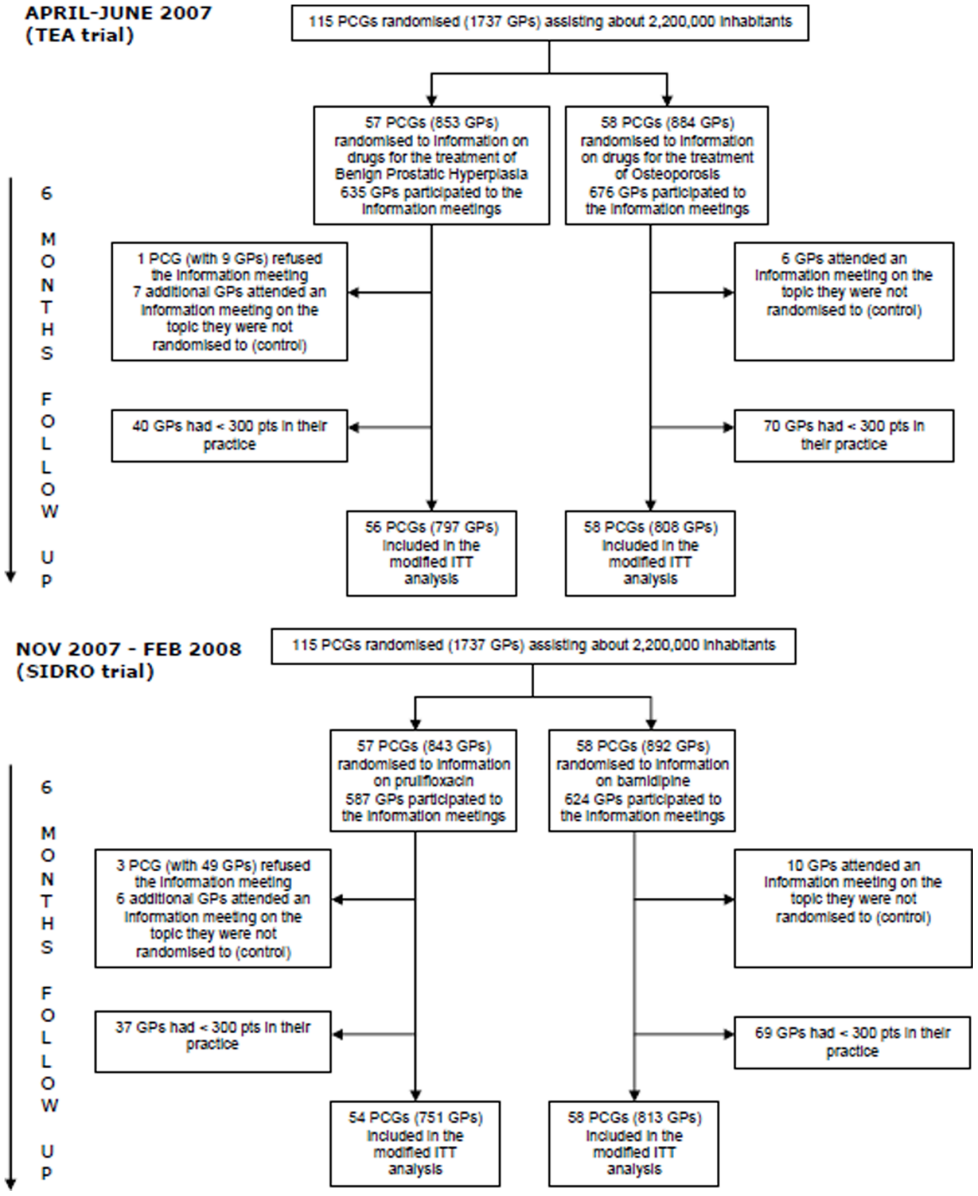
Flow-charts of the two cluster RCTs.

Primary Care Groups (PCGs) are associations of about 10–15 general practitioners (GPs) constituted to improve the continuity and local provision of primary health care: PCGs were the unit of randomisation in the two cluster trials. All PCGs of the five provinces were asked to participate in two rounds of small group interactive information meetings (one in spring and one in autumn 2007). Within each round of visits, PCGs were randomised in two groups: those receiving information about a topic (A) and those receiving information about a different topic (B), unrelated to (A) (Fig. 1). Groups were compared using for each topic a set of specific prescribing outcomes: group receiving A compared to group “not receiving A” (actually receiving B) and vice versa, so two sets of comparisons were performed in each trial. Each group also received the presentation and discussion of 2 clinical scenarios for each topic and prescribing data concerning the group prescription vs the average prescription in the Local Health Authority (LHA). Feedback on individual prescriptions was avoided to favour a group evaluation rather than flattening the discussion on “who has prescribed what”.

The two RCTs had the following distinctive features:

- the 1^st^ RCT (ThErapeutic Area approach or TEA trial), randomised the PCGs in two groups receiving an educational session on a therapeutic topic (treatment of benign prostatic hyperplasia (BPH) or drugs used in the treatment of osteoporosis) using a fairly long information package of 12 pages on benefits and risks of drugs, highlighting lack of superiority data of newer vs older (originator) drugs, availability of different drug classes and of generic drugs;
- the 2^nd^ RCT (SIngle DRug Oriented – SIDRO trial, meaning cider in Italian), compared the same two groups of PCG, in a meeting occurring 3–4 months later, using more focused information material (4 pages information package) on a single “me too” drug recently approved and with rapidly increasing prescriptions: a calcium antagonist (barnidipine) or a fluoroquinolone (prulifloxacin) were the drugs chosen.

In fact these two RCTs offer two different models of approaching doctors’ education: the first approach is disease oriented and closer to thematic courses, requires two ad-hoc trained professionals such as a pharmacist and a physician and tends to provide multiple key messages, often not straightforward for some of the various outcomes considered. The second one is closer to an anti-marketing strategy targeting a single drug, with a pharmacist as the main actor or facilitator of an easy-to-use evidence synthesis, and has more clear-cut outcomes based on prescribing difference of a single drug.

Though the main objective was to estimate the impact of such intervention on the prescribing profile of the various drugs, these two consecutive trials were also designed to allow us to consider differences (in terms of feasibility, acceptability and magnitude of effect) between the two approaches: either a more articulated and “therapeutic area” oriented approach, or a more focused “single drug” approach. They are jointly described in this paper to discuss and contrast these two different approaches, implemented using the same methodology.

The four information packages (the 2007 issues available at http://assr.regione.emilia-romagna.it/it/servizi/pubblicazioni/collane-cessate/archivio-pacchetti/intro) were developed in a clear, simple and appealing format, first reviewing the available evidence, then critically appraising the most relevant or largest among available studies, providing information on absolute benefits and harms of the chosen drugs and some contextual information (e.g. prescribing data and regulatory issues). [22] The idea was to “just present information”, without recommending which of the addressed drugs should be prescribed. In particular, during the outreach visits, available evidence on targeted drugs was presented, highlighting the difference between a statistically significant effect on outcomes of questionable clinical relevance, and their meaning expressed in absolute terms (NNT/NNH, absolute reduction in risks, etc).

The small group meetings were organised by the Departments of Primary Care within each LHA and were part of a continuing medical education program (providing CME credits). They were led by a trained pharmacist, supported by a “referent” GP delegate by his/her PCG who had the main role to stimulate the “inter pares” discussion using *ad-hoc* clinical scenarios and a problem-based approach. Table 1 synthesizes the main components of the multifaceted intervention.

**Table 1:** The main components of the multifaceted educational intervention.

SETTING	bi-annual small group meetings (each lasting 3–4 hours) with a Primary Care Group (PCG)
TOOLS	ad hoc prepared information packages (pharmacists/referent GPs receive one three-day/half aday courses on the contents of that material); presentation and discussion of real life clinicalscenarios; prescribing reports for each PCG comparing with local averages
METHODS	educational outreach visits (small group meetings); audit & feedback; problem-based learning:“we present to you the available evidence and some context, you decide”
ACTORS	a pharmacist acts as facilitator, presenting the evidence-based information packages andcommenting on drug utilization data of PCG; a general practitioner acts as referent peerpresenting real life clinical scenarios to be discussed in the light of available evidence

Approximately one pharmacist per 5–10 PCGs (80–150 GPs) was trained through one four-day intensive course (36 hours) on EBM methodology, a three-day course (about 20–22 hours) for each topic in TEA trial (osteoporosis and BPH) and 1 day course for each single drug in SIDRO trial. One GP for each PCG (referent) received a half-day training module on each topic their practice group had been randomised to. Pharmacists were assigned to each PCGs before randomization.

Stratified randomisation was carried out within each Local Health Authority (LHA), using the number of assisted population per PCG (under or over the mean for that LHA) as stratifying factor. Computer generated random numbers were used to assign PCGs to either group, in order to get the minimum possible difference between groups in terms of number of assisted population per PCG.

### Outcomes

Main outcomes were referred to differences in NHS prescription of drugs under scrutiny (expressed as DDD per thousand patients) during the six months after the educational intervention, with a parallel comparison of individual doctors who had received the specific information topic versus those who received the other one, adjusted for the cluster effect. Baseline imbalances were taken into account calculating the change from baseline in the prescribing rate. Secondary analyses with 12, 24 and 48 months of follow-up were also performed, to assess the persistence of effects in absence of reminders.

Specifically, the TEA trial had four primary outcomes and 11 secondary outcomes (see results section). Some outcomes were designed to evaluate a shift toward off-patent drugs (such as tamsulosin and terazosin for BPH or alendronate for osteoporosis) which were also those with more robust evidence. In general, a prescribing reduction was expected since evidence-based data show that prescription of drugs in those therapeutic areas may be excessive.

In the SIDRO trial a reduction was expected for both the 2 two main outcomes (one for each arm), representing the prescription of me-too drugs.

All data were collected through a regional routine data collection system, containing information (including date of prescription) about all prescriptions written by each doctor to residents of Emilia-Romagna.

In addition to prescribing outcomes, a questionnaire exploring doctors’ knowledge on the addressed topics and attitudes on the information materials was distributed before and after the information meetings. Questionnaires assessing knowledge were specifically designed for each topic and were nominal' (for receiving CME credits), whereas those assessing attitudes were anonymous for a more unbiased evaluation of opinions on completeness, balance and usefulness of the information.

### Statistical considerations

From a sample of 40 clusters (about 600 general practitioners) and using a dedicated software (Cluster Randomisation Sample Size Calculator version 1.0.2, Health Services Research Unit, Aberdeen University) we estimated the intracluster correlation coefficients (ICC) and the sample size that would have been required to see a difference of 10–15% in the prescription of the specific drugs, assuming an average cluster (PCG) size of 15. [25].

With the recruitment of 115 PCGs, and using correction for multiplicity considering primary outcomes of both trials, we had 80% power to detect a 9–15% difference for the various drugs considered.

Doctors' prescriptions were analysed according to the randomisation scheme, independently of their participation in the outreach visits. A few exceptions leading to a modified intention-to-treat analysis (m-ITT) are described below. In particular, the following were excluded from the main analyses:

- general practitioners with fewer than 300 patients; these could have abnormal rates in the prescription of specific drugs if people using these drugs were over-represented among their patients, distorting the trial results; moreover, their practice populations may rapidly grow over time, since they are usually young doctors beginning their activity (in fact no prescribing reports are produced for these GPs since they would not be reliable);
- general practitioners who participated in a different information meeting (different topic) from the assigned one because they had moved to a different PCG after randomization. Since these cases occurred mostly for administrative reasons (re-arranging of PCGs at LHA level) rather than through doctors’ choice, we judged that this specific modification to the ITT would not limit generalizability, whereas keeping those doctors in the original randomization scheme even if receiving a different topic could have led to biased results;
- one PCG refused its consent to participate.

Generalised linear models (random effects models) were used to correct primary outcomes for baseline imbalances in the prescription of targeted drugs.

STATA version 11 was used for data analysis.

### Ethics approval

The two RCTs have been approved by each of the Ethics Committees of the Local Health Authorities (Parma, Reggio Emilia, Modena, Bologna, Forlì) involved in the research. Randomized doctors were not asked to sign an informed consent form: their participation to the information meetings was part of a compulsory program set by the Local Health Authorities. Prescribing data are routinely collected and informed consent is not needed for their collection and analysis. Ethics Committees were of course aware of the recruitment procedure: one of the Committees (Modena) declared that a formal approval was not even necessary for the reasons described above.

### Protocol publication and registration

The protocol has been published in a peer-reviewed journal and is openly accessible at http://www.biomedcentral.com/content/pdf/1472-6963-7-158.pdf.

Even if not strictly necessary (having randomized general practitioners and not patients) we registered the trials on the ISRCTN register (trial registration n.: ISRCTN05866587).

## Results

### First RCT: ThErapeutic Area approach (TEA) trial: Information on drugs for the treatment of benign prostatic hyperplasia (arm 1) and drugs for the treatment of osteoporosis (arm 2)

The flow charts of cluster participants is shown in Figure 1. All 115 PCGs, corresponding to 1737 GPs assisting about two millions inhabitants, were randomised. A total of 1605 GPs (92.4%) were included in the m-ITT analysis, excluding 110 GPs (6.3%) who had less than 300 subjects in their assisted population, one PCG (with 9 GPs –0,5%) refusing to participate to the study, and 13 single GPs (0,7%) who had moved to a different PCG after randomisation. A total of 1311 of the included GPs (81.7%) participated in the information meetings (per protocol population).

Table 2 shows GPs’ mean baseline prescription of drugs considered in the primary outcomes. Limited imbalances between the two groups (less than 5%) had to be considered. Table S1 shows number and characteristics of PCGs and GPs in the ITT analysis.

**Table 2:** TEA trial: GPs’ mean baseline prescription (in the 3 months preceding the outreach visit) of drugs considered in the primary outcomes, expressed as DDD per 1000 patients.

Drugs considered	Group 1: information on drugs for the treatmentof benign prostatic hyperplasia	Group 2: information on drugsfor the treatment of osteoporosis
finasteride	5.35	5.46
dutasteride	4.09	4.30
alfuzosin	9.98	9.66
terazosin	4.64	4.73
tamsulosin	11.05	11.11
alendronate	7.35	7.29
risedronate	3.01	2.98

Table 3 synthesizes differences in change from baseline between intervention and control groups in prescribing outcomes. In the ITT analysis, one BPH-related primary outcome showed a statistically significant reduction: specifically, the ratio between alpha blocker drugs alfuzosin (still under patent) and tamsulosin+terazosin (off patent) decreased by 8.5% (p = 0.03; intracluster correlation coefficient or ICC of almost 0), suggesting a shift towards off patent drugs, as expected. The other BPH-related primary outcome, prescription of finasteride+dutasteride did not reach statistically significant differences (ICC = 0.06).

**Table 3:** TEA trial: differences in DDD per 1000 patients of prescribed drugs (intervention vs control: 1605 included GPs).

	Absolute mean prescriptionduring follow-up in the ‘intervention group’ (DDD per 1000assisted population/day)	Absolute difference atfollow-up vs control group (change from baselineexpressed as DDD per 1000 assisted population/day)	Relative % variationvs control: m-ITT(95% CI)
**Primary outcomes (arm 1: BPH)**			
finasteride+dutasteride	6.61	0.24	3.6% (−2.9 to 10.2%)
alfuzosin vs tamsulosin +terazosin †	0.71 (ratio)	−0.06	−8.5% (−16.9 to −0.7%)
**Secondary outcomes (arm 1: BPH)**			
finasteride	3.60	0.05	1.4% (−6.5 to 9.5%)
dutasteride	3.00	0.18	5.8% (−2.6 to 14.3%)
alfuzosin	6.82	−0.11	−1.7% (−8.9 to 5.6%)
tamsulosin	7.92	0.28	3.7% (−3.1 to 10.4%)
terazosin	3.21	0.17	5.4% (−4.1 to 14.6%)
**Primary outcomes (arm 2: osteoporosis)**			
alendronate	4.99	<0.01	0.1% (−7.3 to 7.5%)
risedronate	2.03	−0.11	−5.1% (−15.3 to 5.6%)
**Secondary outcomes (arm 2: osteoporosis)**			
ibandronate	0.48	−0.11	−19.6% (−33.9 to −5.4%)
alendronate vs risedronate + ibandronate §	2.89 (ratio)	0.27	10.4% (−3.5 to 24.6%)
strontium ranelate	0.59	0.03	4.8% (−12.9 to 21.0%)
raloxifene	0.13	<0.01	0.4% (−26.9 to 27.5%)
calcium	1.83	−0.11	−5.4% (−15.6 to 5.4%)
vitamin D	1.72	−0.06	−3.5% (−12.4 to 5.3%)

† 56 physicians who had not prescribed tamsulosin or terazosin could not be included in the calculation since this is a ratio.

§ 300 physicians who had not prescribed risedronate or ibandronate could not be included in the calculation since this is a ratio.

As for osteoporosis related outcomes, no primary outcome showed a statistically significant reduction in the ITT analysis (ICC for alendronic acid and risedronic acid were 0.04 and 0.31, respectively);

One secondary outcome, prescription of ibandronic acid, showed a statistically significant reduction (−19.6%; p = 0.01; ICC = 0.002), although its prescription was very limited in absolute terms. This drug was shown to lack evidence on primary prevention of osteoporotic fractures.

**Figure 2:**
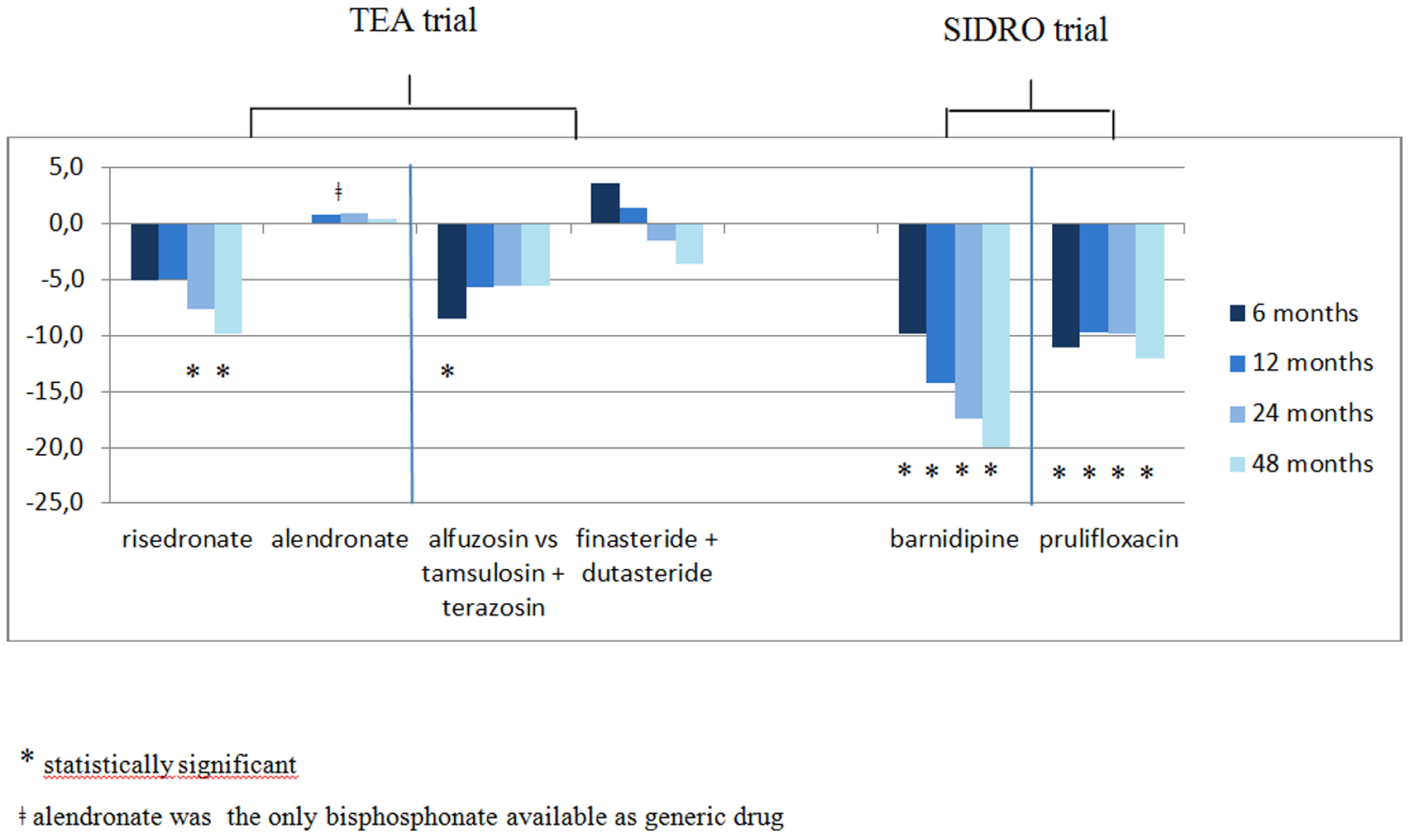
% differences in primary outcomes at 6 months (main analysis), 12, 24 and 48 months.

Fig. 2 provides a clear representation of results also related to longer term follow-ups (after 12, 24 and 48 months), allowing for an evaluation of persistence of effects: similar direction to 6-month results are showed, except for combined prescription of finasteride and dutasteride in BPH. Ratio between alpha blocker drugs alfuzosin and tamsulosin+terazosin lost statistical significance after 12 months. Surprisingly, prescription of risedronate in osteoporosis was further reduced and was statistically significant after 24 and 48 months (−7.6%, p = 0.02; and −9,8%, p = 0.03).

### Second RCT: SIngle DRug Oriented (SIDRO) trial: A new antibiotic and a new antihypertensive (a calcium-channel blocker): prulifloxacin (arm 1) and barnidipine (arm 2)

In this subsequent trial, all 115 PCGs, corresponding to 1735 GPs, were randomised (two general practitioners retired in the period between the first and the second trial – Fig. 1).

A total of 1564 GPs (90.1%) were included in the ITT analysis, excluding 106 GPs (6.1%) who had fewer than 300 subjects in their assisted population, three PCGs (with 49 GPs –2,8%) refusing the small group meeting, and 16 single GPs (0,9%) who had moved to a different PCG after randomization receiving the “control” information (Fig. 1); 1211 of the included GPs (77,4%) participated in the information meetings (per protocol population).

Table 4 shows GPs’ mean baseline prescription for the drugs of interest. Limited imbalances between the two groups (about 6%) had to be considered. Table S2 shows number and characteristics of PCGs and GPs in the ITT analysis.

**Table 4:** SIDRO trial: GPs’ mean baseline prescription (in the 3 months preceding the outreach visit) of considered drugs, expressed as DDD per 1000 patients.

Drugs considered:	Group 1: information on prulifloxacin	Group 2: information on barnidipine
prulifloxacin	0.19	0.18
barnidipine	1.07	1.06

Table 5 synthesizes differences in change from baseline between intervention and control groups in prescribing outcomes related to the prulifloxacin and barnidipine information meeting. In the modified ITT analysis, both the primary outcomes showed a statistically significant reduction of about 10%: prulifloxacin −11.1% (p = 0.04; ICC = 0.05); barnidipine −9.8% (p = 0.02; ICC = 0.08). This suggests that information focused on a single drug rather than on more complex therapeutic strategies can be successful in changing doctors’ behaviours and this result is stable or even increasing over time (up to 4 years); however, caution is needed in interpreting these data, considering the limited prescription of these drugs in absolute terms (in particular of prulifloxacin).

**Table 5:** SIDRO trial: differences in DDD per 1000 patients of prescribed drugs (intervention vs control).

prescribing outcomes(primary)	Absolute mean prescription duringfollow-up in the ‘intervention group’(DDD per 1000 patients)	Absolute difference at follow-up vscontrol group (change from baselineexpressed as DDD per 1000 patients*)*	m-ITT (95% CI)(1564 included GPs)
prulifloxacin	0.17	−0.02	−11.1% (−0.5 to −22,2%)
barnidipine *	1.94	−0.21*	−9.8% (−1.9 to −18.2%)

*a lower increase was observed in the intervention group.

Results after 12, 24 and 48 months showed, surprisingly, a progressively larger reduction in the prescription of barnidipine, doubled after 48 months, and similar reductions in the prescription of prulifloxacin, suggesting that the effect of the intervention might have persisted over time (Fig. 2).

### Assessment of doctors’ knowledge and attitudes

The majority of doctors could identify the correct answers to several questions and to improve their baseline knowledge after the intervention, although answers were given collectively in most of the information meetings, limiting the usefulness of such analysis. For this reason, those results are not shown.

As for doctors’ opinions on completeness and usefulness of the information received, the vast majority of general practitioners evaluated it as “very much” or “quite”, as shown in Table 6. Even if the more articulated information presented in the first RCT was associated with less evident changes in the prescribing outcomes, compared with the more focused information of the second RCT (as described above), such information was more appreciated by doctors: more than 90% considered information packages in the first RCT either “very” or “quite” useful compared to 72–75% in the second RCT.

**Table 6:** Participants’ opinions about usefulness of information received (question: Do you think the information you have received will be useful?).

Information package	Very much	Quite	Little	Not at all	No answer
**Osteoporosis**	42.5%	53.5%	1.8%	0.1%	2.1%
**BPH**	29.7%	62.3%	4.5%	0.9%	2.6%
**Prulifloxacin**	31.0%	43.6%	19.1%	3.1%	3.2%
**Barnidipine**	37.3%	35.8%	18.2%	6.3%	2.3%

## Discussion

These two cluster RCTs confirm that pharmacists’ outreach visits can be an effective way of promoting evidence-based prescribing in a large NHS setting and show that effects can persist in the long-term (up to 4 years). They are also the largest studies done in terms of number of doctors involved. A cluster design was used since group meetings are held regularly (at least twice a year) with Primary Care Groups in the intervention areas and because they represent a natural setting where to deliver information, stimulate analysis and discussion through a problem-based approach. [26]–[27].

In the first RCT (TEA trial) in which information materials addressed a therapeutic area and had broader clinical scope and extent (drugs in the treatment of benign prostatic hyperplasia/osteoporosis), only one of the four predefined primary outcome showed a statistically significant reduction (prescription of alfuzosin compared to tamsulosin and terazosin in benign prostatic hyperplasia). The fairly ambitious quantitative hypothesis to be tested (10−15% difference in doctors' prescriptions) was thus substantially not demonstrated, in spite of the involvement of about 1800 general practitioners. Among the other three primary outcomes in the first RCT, one (prescription of risedronate) has become statistically significant after two years of follow-up, further reducing its prescription in the intervention group, and this finding is not easy to interpret; another primary outcome (prescription of alendronate) was affected by its availability as generic drug just before the start of the trial, as well as by its better assessed benefit-risk profile compared to the other bisphosphonates; this may have reduced the potential to show a difference between the two groups. As for bisphosphonates, the prescription of ibandronate (a secondary outcome) showed a reduction in the intervention group, but this drug was little prescribed.

Conversely, in the second RCT (SIDRO trial) which concerned information on a single drug, both primary outcomes showed clear and statistically significant prescribing reductions (10% or more) lasting a long time. Although doctors generally appreciated the therapeutic area approach used in the first RCT - addressing both diagnostic and therapeutic questions in a broad range of clinical scenarios - more than the more focused single drug approach, the latter seems to have affected doctors’ prescribing behaviour more clearly.

These studies confirm findings from previous RCTs conducted mostly in Northern Europe and North America, showing that pharmacists delivering evidence-based information and prescribing data through outreach visits may influence doctors’ prescribing behaviour in the short term. Our findings add that positive results are mostly obtained with an intervention focused on a single drug, suggesting that the more complex the information, the more complex and the less likely the related behaviour/prescribing change. Contrary to the findings of previous educational intervention trials, we found high persistence of the effects (measured initially as stated in the study protocol at 6 and 12 months, and in subsequent periods up to 48 months) even in absence of reminders. This result was particularly sharp for barnidipine, a calcium antagonist possibly suffering from the competition of many drugs in the same class.

The evaluation of educational interventions is fairly complex given the many different variables in play. [28] Among these, we did not consider patient demand as a main driver of drug prescription, giving priority to feasibility and replicability of the intervention. However, the pragmatic nature of these RCTs and their large-scale implementation in a natural setting strengthen the external validity of the results.

The context may determine the success of these educational programs, by hindering or facilitating the implementation of small group meetings. In this regard, general practitioners from Friuli Venezia-Giulia (another region in Northern Italy), initially supposed to take part in the RCTs, did not in the end do so because their representatives and the Regional Health Authority disagreed. Moreover, one more RCT was originally planned in Sardinia (a large island in Southern Italy), to compare three different strategies of information delivery to solo general practitioners: [21] this trial did not take place for similar reasons as in Friuli Venezia-Giulia. Involvement of doctors’ organizations and strong endorsement of Health Authorities are crucial in implementing such an anti-promotional intervention; doctors may see these interventions as top-down, cost-saving approaches.

In conclusion, the confirmation of a positive impact of independent information programs in a large-scale natural setting, in particular when information on single drugs is provided, can be considered as a fairly relevant finding and the persistence of the observed differences for up to 4 years is quite a new one. If the organizational environment is “good enough” and a strong endorsement from Health Authorities (with the sole aim to promote public health optimizing treatments) is assured, information programs led by National Health Systems Agencies may provide a critical alternative to industry-led information to physicians. Future studies should focus on assessing whether positive findings are driven by how information is delivered (small groups versus one-to-one meetings) and presented, evaluating different formats and ways of presenting the actual magnitude of benefits and risks of drugs and other health interventions.

## Supporting Information

Table S1 **TEA trial: number and characteristics of PCGs and GPs in the ITT analysis.**
(DOCX)

Table S2 **SIDRO trial: number and characteristics of PCGs and GPs in the ITT analysis.**
(DOCX)

Table S3 **Primary data for the TEA trial.**
(XLSX)

Table S4 **Primary data for the SIDRO trial.**
(XLSX)
