# Supplementary material for: Long Term Effectiveness on Prescribing of Two Multifaceted Educational Interventions: Results of Two Large Scale Randomized Cluster Trials
Source: PLoS One. 2014 Oct 17;9(10):e109915. doi: 10.1371/journal.pone.0109915 (PMC4201458; doi:10.1371/journal.pone.0109915)
Supplement: Table S2 — SIDRO trial: number and characteristics of PCGs and GPs in the ITT analysis. (DOCX) [file pone.0109915.s002.docx]

*Table S2. SIDRO trial: number and characteristics of PCGs and GPs in the ITT analysis*

|  | Group 1: information on prulifloxacin | Group 2: information on barnidipine |
| --- | --- | --- |
| n. PCGs included in the ITT analysis | 54 | 58 |
| mean n. GPs per PCG | 14 | 14 |
| n. GPs included in the ITT analysis | 751 | 813 |
| mean n. assisted population per GP | 1222 | 1193 |
| mean n. assisted population per PCG | 17058 | 16672 |
| mean GP age | 55 | 56 |
| n. (%) GPs included who participated in the information meetings | 587  (78.2%) | 624  (76.8%) |
